# General practice management of rotator cuff related shoulder pain: A reliance on ultrasound and injection guided care

**DOI:** 10.1371/journal.pone.0227688

**Published:** 2020-01-13

**Authors:** Josh Naunton, Christopher Harrison, Helena Britt, Terrence Haines, Peter Malliaras

**Affiliations:** 1 Physiotherapy Department, School of Primary and Allied Health Care, Faculty of Medicine Nursing and Health Sciences, Monash University, Victoria, Australia; 2 Menzies Centre for Health Policy, Sydney School of Public Health, Faculty of Medicine and Health, The University of Sydney, Sydney, NSW, Australia; 3 Sydney School of Public Health, Faculty of Medicine and Health, The University of Sydney, Sydney, NSW, Australia; 4 School of Primary and Allied Health Care, Faculty of Medicine Nursing and Health Sciences, Monash University, Victoria, Australia; Cleveland Clinic, UNITED STATES

## Abstract

**Objective:**

To describe general practitioner's (GP's) current management of rotator cuff related shoulder pain (RCRP) in Australia and identify if this is consistent with recommended care and best available evidence. The secondary aim was to determine if GP management of RCRP changed over time.

**Methods:**

Data about management of RCRP by Australian GPs was extracted from the Bettering the Evaluation of Care of Health program database over its final five years (April 2011-March 2016). Patient and GP characteristics and encounter management data were extracted. Results are reported using descriptive statistics with point estimates and 95% confidence intervals. A secondary analysis over a 16 year period (2000–2016) examined management data for RCRP in four year periods.

**Results:**

RCRP was the most common shoulder condition managed by GPs at 5.12 per 1,000 encounters; and at an estimated 732,000 times nationally in 2015–2016. Management rate was higher among male patients (5.5 per 1000 encounters c.f. 4.8 for female patients) and was highest in the 45–64 year old age group (8.6 per 1000). RCRP was most frequently managed with medications (54.7%), steroid injection (19.5%) followed by non-steroidal anti-inflammatory drugs (NSAIDs) (19.1%). Imaging was ordered for 43.4% (ultrasound 41.2% and x-ray 11.6%) of all RCRP presentations (new and returning). Over half (53.0%) of new RCRP presentations were referred for ultrasound imaging. In the 16 year period 2000–16 ultrasound imaging more than doubled from 19.1% to 41.9% of management occasions. In parallel, prescribed steroid injection increased from 9.8% to 19.7%.

**Conclusion:**

The usual care provided by GPs for RCRP relies on the use of ultrasound and steroid injection. This is not consistent with recommended care and clinical guidelines that recommend these are delayed until after 6–12 weeks of NSAID medication, exercise and activity modification. There has been a significant increase in the rate of steroid injection and ultrasound imaging, which may be due in part to policy change.

## Introduction

Shoulder pain is the third most common musculoskeletal condition given by patients as a reason for encounter to general practitioners (GPs) in Australia, described at 1.2% of all GP encounters [1]. Rotator-cuff related pain (RCRP) is widely considered the most common cause of shoulder pain [2, 3], specifically accounting for 70% of cases in Dutch general practice [4]. RCRP is a clinical diagnosis and presentation that includes pain localised to the anterolateral shoulder, typically aggravated with overhead activity. There is typically pain with active or resisted shoulder abduction, a painful arc into shoulder abduction and passive glenohumeral joint range is maintained [5]. The rotator cuff tendons and subacromial bursa are thought to be the key sources of pathology in RCRP [6] and pathology includes tendinosis (inferior quality and disorganised collagen), partial and full thickness tears, and thickening of the subacromial bursa [6]. Structural pathology, even tears, are common in people without symptoms so are not the source of pain per se [7]. Altered biochemical pain signalling (e.g. substance P) is considered an important pain mechanism rather than structure in itself [8]. Excessive activity (beyond the adaptive potential of the tendon) is considered a key aetiological factor, moderated by intrinsic factors including scapular muscle function, older age, higher BMI, and poor metabolic health (e.g. elevated cholesterol) [9]. RCRP is more common as people age [9, 10]. It can severely limit work and daily functions, including dressing and eating; and up to 40% of people affected will experience ongoing disability beyond 12 months [9, 10].

Exercise is recommended by RCRP practice guidelines, along with rest from provocative activities, and medications (analgaesic or anti-inflammatory drugs) [3, 8, 11–13]. Between 65% and 80% of people with RCRP, even with rotator cuff tears, recover (large improvement or complete recovery) with exercise [14]. There are no clinically important benefits of surgery over exercise for RCRP [15–18]. Given it is less expensive and there are less serious risks, it is logical that exercise is recommended prior to surgery. Despite this, single state data from Western Australia showed a doubling and tripling in population-adjusted rates and costs of surgeries for RCRP between 2001 and 2013 respectively [19] and there are similar reports of increasing surgical rates in the United Kingdom (UK) and America [20–22].

As primary care clinicians, GPs are likely to be the first clinical contact for many people with RCRP. GPs are encouraged to take a patient centred approach with a focus on education and advice regarding activity modification, including referral to physiotherapy [11–13]. If analgaesia is required, paracetamol and NSAIDs are recommended as first line care, either individually or in combination if paracetamol alone is not helpful [5, 11, 13]. Guidelines published from Australia [8], America [23], the UK [11], Italy [24] and the Netherlands [12] agree that, in the absence of red flags, imaging is not recommended for people with non-traumatic RCRP until they have trialled 6 weeks or more of first line recommended treatment [3, 5, 8, 12, 13].

Despite guideline recommendations, the management for musculoskeletal conditions is known to vary widely amongst primary care practitioners [25–30]. A recent survey of Australian GPs (n = 611) and rheumatologists (n = 70) found that, based on a clinical vignette of non-traumatic RCRP, 69% of GPs indicated their management would include an x-ray at first presentation prior to first line recommended care and 50% of surveyed rheumatologists considered this appropriate primary care [31]. In addition, 82% of GPs indicated their management would include an ultrasound and 60% of rheumatologists considered this appropriate primary care [31]. These findings suggest there is a large disconnect between recommended and actual care for RCRP amongst Australian GPs.

Unnecessary imaging for RCRP contributes to escalating healthcare costs related to diagnostic imaging and may be harmful for patients. Imaging costs for all shoulder ultrasound and x-ray were $60 million and $23 million respectively (2018–2019) [32, 33]. The issues of unnecessary imaging and low value care are well recognised, as evidenced by global campaigns such as Choosing Wisely. The Choosing Wisely campaign commenced in 2012 and calls for action to reduce unnecessary healthcare that has low value or has been proven to be ineffective, including unnecessary referrals for ultrasound and x-ray [34, 35]. Whether Choosing Wisely has been effective in reducing unnecessary RCRP imaging among Australian GPs is not known.

This study aims to describe current GP practice in managing RCRP (management frequency, types of treatment, referral practices, associated GP and patient demographics) utilising data available from the Bettering the Evaluation and Care of Health (BEACH) program [1, 36]. A secondary aim was to determine if there was substantial change in GP practice between 2000–01 and 2015–16. Understanding practice patterns in GP management of RCRP will assist in the development of strategies to reduce any apparent evidence to practice gap between high quality recommended care and clinician practice.

## Methods

The analysis was performed on data from the BEACH study, a cross-sectional paper based national study of general practice clinical activity. Nationally representative samples of about 1,000 active GPs participated each year from April 1998 to March 2016. Participating GPs each recorded details of 100 consecutive encounters of all types, with consenting patients. Encounter details recorded included: patient characteristics; patient reasons for encounter (RFEs); problems managed (including new/follow-up status); medications (including prescribed, supplied and advised for over-the-counter purchase); therapeutic procedures; clinical treatments (such as advice and counselling); referrals; pathology and imaging tests ordered. Each management action is linked by the GP to the individual problem managed. New problems were defined as the first presentation of a problem, including the first presentation of a recurrence of a previously resolved problem, but excluding the presentation of a problem first assessed by another provider.

Medications were classified according to the Anatomic Therapeutic Chemical (ATC) classification [37, 38]. Patient RFEs, problems managed and all non-pharmacological managements were classified according to the International Classification of Primary Care—Version 2 (ICPC-2) [39] but were coded more specifically to the Australian GP terminology ICPC-2 PLUS [40]. Patient relative socioeconomic status was determined by the Index of Relative Socio-Economic Advantage and Disadvantage (IRSAD) [41] based on patient residential postcode. A patient’s language background was determined by whether the primary language spoken at home was English or not. Patient Indigenous status was determined by patient self-identification as Aboriginal and/or Torres Strait Islander. Practice rurality was determined by the Australian Statistical Geography Standard [42]. S1 Table lists how we defined RCRP, acute rotator cuff related injuries, general shoulder pain and all management actions.

### Analysis

We first examined the management rate of each of the shoulder pain categories (defined in S1 Table) across the final five years of BEACH data (April 2011-March 2016). We extrapolated the management rate by the number of GP items of service claimed through Medicare (Australia’s universal health scheme) for 2015–16 (143 million) to estimate how often each condition was managed by GPs in 2015–16. We sought to identify GP and patient related factors at the univariate level associated with rate of presentation for RCRP (reported as management rate per 1000 encounters), then performed multivariate logistic regression to determine the independent factors associated with RCRP management.

The multivariate logistic regression was performed using the survey logistic procedure in SAS 9.4. Robust variance estimates were again employed to take into account the clustered sampling of data within this study. All patient and GP characteristics were initially placed in each model and then backwards elimination was used to reach parsimonious models using an alpha 0.05 as a retention point.

We examined how RCRP was managed by GPs (the types of interventions implemented by the GPs, reported as proportion (%) of all RCRP problems managed with at least one of the selected management actions) independently for new cases of, and for follow-up consultations of RCRP. Finally, we examined frequency of GP management of RCRP (reported as proportion (%) of all RCRP problems managed with at least one of the selected management actions) over the 16 year period (2000–04, 2004–08, 2008–12, 2012–16).

The BEACH study has a cluster design with each GP having 100 patient encounters clustered around them. Survey procedures in SAS 9.4 were used to calculate robust 95% confidence intervals (95% CIs), which account for the cluster sample design, and are reported around the point estimates. Two point estimates were considered significantly different if the 95% CIs did not overlap. This estimate of difference is more conservative than the 5% level [43], which reduces the risk of Type I error and increases the risk of Type II error.

During the data collection period assessed by this study (April 2000-March 2016), the BEACH program was approved by the Human Research Ethics Committee of the University of Sydney (ethics protocol reference, 2012/130) and by the Australian Institute of Health and Welfare ethics committee for the years they collaborated on this project (2000–11). In compliance with the ethics protocol for the BEACH study, each patient was provided with a written patient information card and then required to give verbal informed consent. If the patient refused, details of the encounter were not recorded. Data collected was not sufficient to identify individual patients.

## Results

From April 2011 to March 2016, 4,881 GPs took part in the BEACH study, recording details of 488,100 patient encounters. Shoulder pain was managed at least once at 13.84 per 1,000 encounters (Table 1). The most common was RCRP (5.12 per 1,000 encounters). This extrapolates to approximately 732,000 (95% CIs: 697,000–766,000) occasions of GP management of RCRP per year nationally in 2015–16. This was followed by other shoulder pain (4.69 per 1000) then acute rotator cuff related injuries (3.04 per 1000), arthritis of the shoulder (0.89 per 1000) and fracture of the shoulder (0.11 per 1,000). Overall, a similar number of shoulder pain problems were new cases (5.71 per 1,000 encounters) as were problems seen previously (6.03 per 1,000 encounters).

**Table 1:** Rate of shoulder pain problems management per 1,000 GP-patient encounters (n = 488,100), April 2011-March 2016, by problem status.

	New cases Rate per 1,000 encounters (95% CIs)	Seen previously Rate per 1,000 encounters (95% CIs)	Status not recorded Rate per 1,000 encounters (95% CIs)	Total Rate per 1,000 encounters (n = 488,100) (95% CIs)
**Rotator cuff related pain**	2.26 (2.11–2.41)	2.20 (2.06–2.35)	0.65 (0.57–0.73)	**5.12** **(4.88–5.36)**
**Acute rotator cuff related injuries**	1.35 (1.23–1.46)	1.28 (1.17–1.39)	0.41 (0.35–0.47)	**3.04** **(2.86–3.22)**
**Arthritis of shoulder**	0.19 (0.15–0.23)	0.57 (0.49–0.64)	0.13 (0.10–0.17)	**0.89** **(0.80–0.98)**
**Fracture of shoulder**	0.04 (0.02–0.05)	0.06 (0.03–0.08)	0.02 (0.01–0.03)	**0.11** **(0.08–0.14)**
**Other shoulder pain**	1.88 (1.74–2.01)	1.92 (1.78–2.06)	0.90 (0.80–0.99)	**4.69** **(4.47–4.91)**
**Total shoulder pain**	**5.71** **(5.47–5.94)**	**6.03** **(5.77–6.28)**	**2.11** **(1.96–2.26)**	**13.84** **(13.45–14.24)**

Table 2 examines the characteristics of patients and GPs at RCRP encounters. RCRP was managed at a significantly higher rate at male patient encounters (5.5 per 1000) than at female encounters (4.8 per 1000). The management rate of RCRP increased with patient age group to peak at 45–64 years (8.6 per 1000), then significantly decreased in each of the older age groups. Patients from more disadvantaged areas had a significantly higher management rate of shoulder pain (5.7 per 1000) than those from more advantaged areas (4.8 per 1000).

**Table 2:** Univariate and multivariate patient and GP characteristic specific management rate of RCRP problems per 1,000 encounters April 2011-March 2016.

	Sample size (n)	Characteristic specific Rotator cuff related pain problems managed per 1,000 encounters (95% CIs)	Odds ratios (multiple logistic regression) (95% Cis)
PATIENT CHARACTERISTICS			
**Patient sex *(missing–n)***	*4*,*261*		Not significant
Male	195,991	5.5 (5.2–5.9)	-
Female	287,848	4.8 (4.5–5.1)	-
**Patient age *(missing–n)***	*4*,*146*		P<0.0001
0–14 years	55,289	0.1 (0.0–0.2)	0.048 (0.022–0.104)
15–24 years	39,075	1.5 (1.1–1.9)	0.515 (0.355–0.748)
25–44 years	107,575	4.0 (3.6–4.4)	1.355 (1.033–1.778)
45–64 years	132,027	8.6 (8.0–9.1)	2.865 (2.227–3.686)
65–84 years	123,224	6.3 (5.8–6.8)	2.246 (1.749–2.884)
85+ years	26,764	2.7 (2.1–3.4)	Reference group
**Socioeconomic status**	*10*,*464*		P = 0.0051
Most advantaged	288,605	4.8 (4.5–5.1)	0.871 (0.791–0.959)
Most disadvantaged	189,031	5.7 (5.3–6.1)	Reference group
**Commonwealth health concession card holder**	*41*,*176*		P = 0.0009
Yes	200,495	5.2 (4.8–5.5)	Reference group
No	246,429	5.1 (4.8–5.4)	1.185 (1.072–1.309
**Language background**	*38*,*901*		Not significant
Non-English speaking	38,901	5.7 (4.9–6.6)	-
English speaking	430,863	5.1 (4.8–5.3)	-
**Indigenous**	*48*,*417*		Not significant
Indigenous	8,820	3.6 (2.4–4.9)	-
Non-Indigenous	430,863	5.2 (4.9–5.4)	-
**GP CHARACTERISTICS**			
**Practice location**	*1*,*300*		Not significant
Major city	343,500	4.9 (4.6–5.2)	-
Inner regional	95,800	5.6 (5.1–6.2)	-
Outer regional/remote	47,500	5.5 (4.6–6.4)	-
**GP sex**	*9*,*000*		P<0.0001
Male	287,700	5.7 (5.3–6.0)	Reference group
Female	209,400	4.4 (4.0–4.7)	0.801 (0.722–0.888)
**GP age**	*2*,*900*		Not significant
<45 years	128,300	4.3 (3.9–4.7)	-
45–54 years	143,000	5.5 (5.1–6.0)	-
55+ years	213,900	5.3 (4.9–5.7)	-
**Country of graduation**	*1*,*700*		Not significant
Australian graduate	323,100	5.2 (4.9–5.5)	-
Overseas graduate	163,300	4.9 (4.5–5.3)	-

Male GPs managed RCRP significantly more often (5.7 per 1000) than female GPs (4.4 per 1000), while GPs working in major cities managed RCRP significantly less often (4.9 per 1000) than those in outer regional/remote areas (5.5 per 1000). Younger GPs (aged less than 45 years) had a significantly lower management rate (4.3 per 1000) than their older peers, aged 45–54 years (5.5 per 1000), and 55+ years (5.3 per 1000).

The backwards elimination multiple logistic regression model identified that patient age, socioeconomic disadvantage in the patient’s area of residence, and absence of a health care card were independent predictors of GP management of RCRP. The patient age-group 45–64 years was found to have the highest predictive value of the factors in this model.

Table 3 examines the management actions in percentage terms for new cases of RCRP, old RCRP and all RCRP. Overall, RCRP was managed most frequently with medications (54.7% of all RCRP), which were primarily steroid injections (19.5%) and NSAIDs (19.1%). Imaging tests were the next most frequently used management (43.4%), ultrasound (41.2%) being more frequent than x-ray (11.6%). Referrals to specialist or allied health services were less frequently made (19.2%). Referrals to physiotherapy (12%) accounted for more than half of all referrals, and were more common than referral to surgeons (4.6%) and rheumatologists (0.4%). The least common management action for all RCRP was advice/education/counselling (10.4%).

**Table 3:** Proportion of occasions at which RCRP was managed with at least one of each of the following actions at encounters.

	New RCRP pain N = 1,103 (95% CIs)	Old RCRP pain N = 1,076 (95% CIs)	All RCRP pain N = 2,497 (95% CIs)
**Medication**	**54.4 (51.4–57.4)**	**55.0 (51.9–58.1)**	**54.7 (52.7–56.8)**
NSAID	23.5 (20.9–26.0)	15.8 (13.6–18.0)	19.1 (17.6–20.7)
Steroid–injection	15.8 (13.6–18.0)	22.7 (20.0–25.3)	19.5 (17.8–21.2)
Opioid	6.5 (5.1–8.0)	9.9 (8.0–11.7)	8.0 (6.9–9.1)
Panadol	8.3 (6.7–10.0)	7.0 (5.4–8.6)	7.5 (6.4–8.6)
Steroid–oral	0.4 (0.0–0.7)	0.5 (0.1–0.9)	0.4 (0.2–0.7)
**Advice/education/counselling**	**10.7 (8.8–12.6)**	**10.7 (8.8–12.6)**	**10.4 (9.1–11.7)**
Exercise	1.0 (0.4–1.6)	0.7 (0.2–1.1)	0.8 (0.5–1.2)
Medication	0.2 (0.0–0.4)	1.0 (0.4–1.6)	0.6 (0.2–0.9)
**Procedure**	**15.7 (13.4–17.9)**	**18.8 (16.2–21.4)**	**17.4 (15.7–19.1)**
Physical medicine /rehabilitation	8.0 (6.3–9.6)	5.4 (3.9–6.9)	6.7 (5.6–7.8)
**Referral**	**17.8 (15.4–20.1)**	**20.5 (18.0–23.0)**	**19.2 (17.5–20.8)**
Physiotherapist	12.6 (10.5–14.7)	11.4 (9.5–13.4)	12.0 (10.7–13.4)
Surgeon	3.1 (2.0–4.1)	6.0 (4.6–7.5)	4.6 (3.8–5.4)
Rheumatologist	0.2 (0.0–0.4)	0.7 (0.2–1.3)	0.4 (0.1–0.7)
**Imaging**	**55.1 (52.1–58.1)**	**30.9 (28.0–33.9)**	**43.4 (41.3–45.4)**
Ultra sound shoulder	53.0 (50.0–56.1)	28.8 (26.0–31.6)	41.2 (39.2–43.3)
X-ray shoulder	19.0 (16.6–21.4)	3.7 (2.6–4.8)	11.6 (10.3–12.9)
MRI shoulder	0.5 (0.1–0.8)	0.6 (0.1–1.0)	0.5 (0.2–0.8)
CT scan shoulder	0.2 (0.0–0.4)	0.1 (0.0–0.3)	0.1 (0.0–0.3)

Notes: Missing data removed. For some problems, the GP did not indicate whether the problem was new or old.

NSAID: Non-steroidal anti-inflammatory drugs

There were significant differences in the management of new RCRP and old RCRP. NSAIDs were more likely to be used for new RCRP (23.5%) than for old RCRP (15.8%). Whereas steroid injection was more likely to be prescribed for old RCRP (22.7%) than for new RCRP (15.8%). Imaging was significantly more likely to be ordered for new RCRP (55.1%) than for old RCRP (30.9%). The differences were similar for ultrasound imaging (new RCRP 53.0%; old RCRP 28.8%) and for x-ray (new RCRP 19.0%; old RCRP 3.7%). Although the referral rate did not differ for new and old problems, the rate of referral to surgeons was significantly higher in management of old cases than of new cases. There was no difference in management of new RCRP and old RCRP in advice/education/counselling.

Table 4 examines GP management of RCRP by time period from 2000–16 in four year intervals. There was a 78% increase in the management rate of RCRP over the study period. The rate (per 1000 encounters) increased in a step-wise manner over each four year time period: from 2.98 in 2000–04, to 3.50 in 2004–08, to 4.28 in 2008–12 and to 5.30 in 2012–16.

**Table 4:** GP management of RC related shoulder pain by time period (2000–2016).

	2000–2004 N = 1190 (95% CIs)	2004–2008 N = 1347 (95% CIs)	2008–2012 N = 1685 (95% CIs)	2012–2016 N = 2064 (95% CIs)
Management rate (per 1000 encounters)	2.98 (2.78–3.19)	3.50 (3.26–3.73)	4.28 (4.03–5.52)	5.30 (5.02–5.57)
Medication	57.1 (54.0–60.1)	47.9 (44.9–50.8)	51.0 (48.5–53.6)	54.3 (52.0–56.6)
NSAID	33.6 (30.7–36.5)	22.9 (20.4–25.3)	20.5 (18.4–22.5)	18.4 (16.7–20.1)
Steroid–Injection	9.8 (7.9–11.8)	10.3 (8.3–12.3)	15.8 (13.9–17.8)	19.7 (17.8–21.6)
Opioid	6.6 (5.2–8.1)	6.9 (5.5–8.3)	7.5 (6.2–8.8)	8.1 (6.9–9.3)
Panadol	7.1 (5.6–8.6)	6.7 (5.3–8.1)	7.0 (5.8–8.2)	7.4 (6.1–8.6)
Steroid–oral	0.2 (-0.1–0.4)	0.4 (-0.0–0.8)	0.1 (-0.0–0.3)	0.5 (0.2–0.8)
**Advice/education/counselling**	**13.1 (11.1–15.1)**	**11.4 (9.6–13.3)**	**11.6 (10.0–13.2)**	**9.9 (8.5–11.4)**
Exercise	2.5 (1.6–3.4)	1.9 (1.1–2.7)	2.3 (1.6–3.0)	0.6 (0.2–0.9)
Medication	0.8 (0.3–1.2)	0.4 (0.1–0.8)	0.4 (0.1–0.6)	0.6 (0.3–0.1)
**Procedure**	**25.3 (22.6–28.0)**	**24.3 (21.3–27.2)**	**21.3 (18.9–23.7)**	**17.0 (15.1–18.9)**
Physical medicine/ rehabilitation	12.0 (10.0–14.0)	8.7 (6.9–10.5)	7.5 (6.2–8.9)	6.6 (5.4–7.8)
**Referral**	**18.7 (16.5–21.0)**	**18.2 (16.0–20.4)**	**18.8 (16.8–20.7)**	**19.3 (17.4–21.1)**
Physiotherapist	10.6 (8.8–12.4)	11.5 (9.7–13.3)	11.2 (9.6–12.9)	12.2 (10.7–13.8)
Surgeon	6.1 (4.7–7.6)	4.4 (3.3–5.5)	5.2 (4.1–6.2)	4.5 (3.6–5.4)
Rheumatologist	1.3 (0.7–2.0)	0.6 (-0.1–1.2)	0.5 (0.1–0.8)	0.3 (0.1–0.6)
**Imaging**	**24.2 (21.7–26.7)**	**27.2 (24.7–29.8)**	**35.5 (33.0–37.9)**	**44.1 (41.8–46.4)**
Ultrasound shoulder	19.1 (16.8–21.4)	24.2 (21.7–26.7)	33.1 (30.7–35.5)	41.9 (39.6–44.2)
X-ray shoulder	13.7 (11.6–15.8)	12.1 (10.3–13.9)	12.2 (10.6–13.9)	11.8 (10.3–13.2)
MRI shoulder	0.0	0.0	0.1 (-0.0–0.3)	0.5 (0.2–0.8)
CT scan shoulder	0.0	0.0	0.3 (0.0–0.6)	0.1 (0.0–0.2)

There was a significant increase in likelihood of steroid injection from 9.8% in 2000–04 to 19.7% in 2012–16. In parallel, there was a significant reduction in NSAID prescription from 33.6% to 18.4% of management occasions, and in the likelihood of procedural treatment, from 25.3% to 17.0%. The only other significant change over time was an increase in the rate of imaging ordered from a likelihood of 24.2% in 2000–04 to 44.1% in 2012–16. Ordering of ultrasound imaging increased significantly from 19.1% to 41.9% of cases while there was no significant change in the ordering of x-ray, 13.7% to 11.8%. There was no change over time in advice/education/counselling, referral, paracetamol or opioid prescription rates.

## Discussion

To the best of our knowledge, this the first study to provide detailed information regarding the management of RCRP by GPs in Australia. These data indicate that RCRP is the most frequent type of shoulder pain managed by GPs at an estimated 732,000 GP-patient consultations nationally in 2015–16. This is similar to the number of GP management encounters for knee OA and more than double the number of encounters for lateral elbow tendinopathy [44, 45].

### GP management of RCRP

The area where GP practice was least consistent with recommended care was in relation to imaging referrals. The rate of orders for ultrasound imaging for new RCRP pain was high (53%) but not as extreme as the national survey of GP responses to a clinical vignette scenario for RCRP (82% would order ultrasound) [31]. This provides strong evidence from two sources (GP survey, and GP practice database), that there are high rates of unnecessary imaging for RCRP occurring prior to recommended conservative care. Unnecessary imaging is likely to lead to further unnecessary treatments. For example, people who are diagnosed with a rotator cuff tendon tear on imaging may proceed to surgery [46], despite evidence that management outcomes are equivalent with conservative care, even among people with rotator cuff tendon tears [15–17]. Unnecessary imaging is concerning given increasing popularity of surgery for RCRP. Western Australia data indicate that rates and costs of surgeries for RCRP doubled and tripled, respectively, from 2001 to 2016 [19]. We estimated that based on new RCRP, managed at a rate of 2.26 per 1000 encounters, 143 million GP encounters in 2015–16 and a Medicare Benefits Schedule (MBS) rebate for shoulder ultrasound of $109.10; there is a potential cost saving of up to $18.7 million if GPs follow recommended care.

It is unclear why GPs continue to utilise ultrasound imaging at such a high rate for new RCRP. There may be GP related factors that explain this trend, such as fear of litigation for missing serious pathology, uncertainty regarding clinical (non-imaging) diagnosis and management, or about appropriate care pathways [47, 48]. There is also a certain level of patient preference for imaging as part of expected care [31, 48]. This can be further reinforced by the practitioner’s desire to avoid conflict with patients, heavy reliance on past experience together with clinical judgement and what is currently accepted as standard practice among their peers [48]. Our findings could also be explained by a distrust for current guidelines, lack of clarity or difficulty implementing guidelines into practice in relation to imaging of RCRP [31, 47, 48]. GPs face many time pressures in their typical day and as such, it is possible that GPs are not aware of the guidelines and have little time to devote to seeking out new or updated guidelines. Exploration of the reasons why GPs do not generally adhere to recommended imaging recommendations when managing RCRP is an integral step in understanding and developing strategies to address this clinical behaviour.

There was a low rate of advice/education/counselling management for new RCRP (10.7%) which is in contrast with recommended care guidelines suggesting first line treatment should include advice and education. There was also a low rate of referral of new RCRP to physiotherapy (12.6%) and does not align with earlier findings that three quarters of surveyed GPs in the national clinical vignette study stated they would refer first presentation RCRP to physiotherapy [31]. Reasons for limited advice and exercise intervention, and referral to physiotherapy in the BEACH database data are unclear. It is possible that, while GPs are aware they should refer new cases to physiotherapy (as reflected in the clinical vignette study) [31], in reality the only problems that can be 'referred' to physiotherapy by GPs with costs covered by the MBS are chronic problems. Typically, physiotherapy costs are covered by the patient. This cost may be partly covered by private health insurance, if the patient is insured for 'extras’; but there is no requirement for the GP to write a formal referral. Patients who do not have private insurance cover for 'extras’ may decline being referred due to patient concerns about cost of these services. We do not know how many patients attend physiotherapy following informal referral or recommendation from their GP. Further, uncertainty regarding the efficacy of exercise (recent Cochrane review published after the National survey) [49] may also impact on GPs decision making when managing RCRP. Finally, Australia has a maldistributed GP workforce [50] and a rapidly increasing population attendance rate to general practice [51], so it is possible that GPs lack the time to adequately assess and manage RCRP within their primary care setting [31, 47].

GPs managed RCRP most frequently with steroid injection (19.5%) and/or NSAIDs (19.1%). They were more likely to prescribe NSAIDs (23.5%) and paracetamol (8.3%) at first presentation of RCRP and more likely to manage with steroid injection for old RCRP (22.7%); this is broadly consistent with current guidelines. There is debate about the clinical timing of steroid injections for RCRP, but a majority of clinical guidelines recommend trialling 6–12 weeks of recommended conservative care prior to steroid injection or surgery. Also consistent with this recommendation, only 5% of GPs referred patients with new RCRP for a surgical opinion.

### GP management of RCRP from 2001 to 2016

The significant increase (78%) in management rate of RCRP highlights the potential multiplication of negative outcomes associated with low value care for GP patients in primary care. The increased management rate may be due to increasing prevalence of RCRP within the community or a change in patient expectation of care. They may be more likely to seek care and have a greater expectation of intervention in managing their pain, than to take a wait-and-see approach. The increase in management rate of RCRP by GPs parallels the increase in surgery rates for RCRP over the past decade [19–22].

There was doubling in the rates of ultrasound imaging (19.1% to 41.9%) and steroid injection (9.8% to 19.7%) between the 2000–04 and the 2012–16 period. This is likely to be related to policy change. There was a sharp increase from 2009 onwards, in the rate of steroid injection and to a lesser extent the rate of ultrasound, which showed consistently increasing rates in each four year period. At the same time in the November 2009 MBS update, the GP item numbers and rebate for anatomical guided injections (items 50124 and 50125) were removed [31]. This meant that a rebate could only be claimed if an injection was delivered with ultrasound guidance. The increase in ultrasound imaging suggests that the Choosing Wisely campaign (commenced 2012 and adopted by the Royal Australian College of General Practitioners [RACGP] in 2015) which aimed to reduce unnecessary imaging, had no effect (based on BEACH data) for RCRP through to 2016. It is also possible that improvements in ultrasound technology and accessibility over this period influenced the change in ultrasound referrals practice. This shift in injection practice contrasts with a 2012 Cochrane review that found no additional benefit for ultrasound guided injection over anatomic landmark based injection [52]. The increased rate of steroid injection may also explain the shift in practise away from management with NSAIDs, with a 55% reduction in NSAIDs usage over this time period from 33.6% to 18.4%. Further work should explore the sociocultural factors that may have contributed to increased usage of imaging and steroid injections over time (e.g. patient expectation for a diagnosis and intervention, GP knowledge and resources).

### Demographic characteristics

Our finding that patients aged 45–64 had the highest rate of GP management for RCRP (8.6 per 1000 encounters) is consistent with epidemiological studies demonstrating increasing rates of rotator cuff pathology in middle aged adulthood [2, 4, 53, 54]. Higher management rates for RCRP were found among patients from most disadvantaged socioeconomic status. A multitude of factors may be associated with RCRP and more prevalent in socioeconomic disadvantaged areas, including rates of metabolic disease and mental health issues such as anxiety and depression [55–57]. The higher management rate of RCRP among male GPs than female GPs, identified after adjustment for other GP and patient characteristics, may reflect the higher management rate of musculoskeletal problems (overall) by male GPs in Australia [58, 59].

### Strengths and limitations

The strengths of this study are based on the large and prospectively recorded data set available from the BEACH study, which has been shown to accurately reflect GP activity for population based management of primary care conditions. It is the only national study of GP activity in the world that is continuous, and directly links management decisions and medications to the problem being managed. We were able to compare change in GP management over a 16 year period from 2000–16 and consider the effect of policy change through this time period [1].

When drawing inferences from these results, the limitations of this study should be considered carefully. The data collected for this study is limited to Australian general practice and different results may be found in other settings. The data are cross sectional and are not meant to be construed as population prevalence for the condition reported. Lastly, guidelines are intended to provide recommendations to guide care and there may be individual patient factors that may require clinicians to vary practice from recommendations in the guidelines. The data from this study do not examine why GPs are not following key recommendations.

## Conclusions

In conclusion, it is clear that GP practise in managing RCRP pain is partly consistent with recommended care. However, our results indicate a strong reliance on ultrasound imaging at first presentation and subsequently a high rate of steroid injection. This contrasts the consensus among recommended practice guidelines that emphasise NSAID medications, exercise and activity modification. There remains an ongoing need for collaboration between researchers, clinicians and policy makers to improve quality of primary care practice in the management of RCRP.

## Supporting information

S1 Table Definitions used within manuscript. (DOCX)
