# Supplementary material for: General practice management of rotator cuff related shoulder pain: A reliance on ultrasound and injection guided care
Source: PLoS One. 2020 Jan 13;15(1):e0227688. doi: 10.1371/journal.pone.0227688 (PMC6957186; doi:10.1371/journal.pone.0227688)
Supplement: S1 Table — (DOCX) [file pone.0227688.s001.docx]

**S1 Table. Definitions Used within Manuscript.**

| **Group** | **Term** | **Code** | **Classification system** |
| --- | --- | --- | --- |
| **Rotator cuff related pain** |  |  |  |
|  | Bursitis; shoulder | L92001 | ICPC 2 PLUS |
|  | Rotator cuff syndrome | L92003 | ICPC 2 PLUS |
|  | Tendonitis; shoulder | L92013 | ICPC 2 PLUS |
|  | Tendonitis; supraspinatus | L92016 | ICPC 2 PLUS |
|  | Painful arc syndrome | L92017 | ICPC 2 PLUS |
| **Acute rotator cuff related injuries** |  |  |  |
|  | Injury; rotator cuff | L79041 | ICPC 2 PLUS |
|  | Injury; supraspinatus | L81023 | ICPC 2 PLUS |
|  | Tear; supraspinatus | L81028 | ICPC 2 PLUS |
| **Arthritis of the shoulder** |  |  |  |
|  | Arthritis; shoulder | L92006 | ICPC 2 PLUS |
|  | Osteoarthritis; shoulder | L92007 | ICPC 2 PLUS |
|  | Humeroscapular periarthritis | L92011 | ICPC 2 PLUS |
|  | Rheumatism; shoulder | L92012 | ICPC 2 PLUS |
| **Fracture of the shoulder** |  |  |  |
|  | Fracture; shoulder | L76055 | ICPC 2 PLUS |
|  | Fracture; acromium | L76056 | ICPC 2 PLUS |
|  | Fracture; scapula | L76057 | ICPC 2 PLUS |
| **Other shoulder problem/pain** |  |  |  |
|  | Pain; shoulder | L08001 | ICPC 2 PLUS |
|  | Pain; musculoskeletal; shoulder | L08004 | ICPC 2 PLUS |
|  | Sprain; shoulder | L79021 | ICPC 2 PLUS |
|  | Strain; shoulder | L79025 | ICPC 2 PLUS |
|  | Injury shoulder | L81018 | ICPC 2 PLUS |
|  | Fibrositis; shoulder | L92002 | ICPC 2 PLUS |
|  | Shoulder syndrome | L92004 | ICPC 2 PLUS |
|  | Synovitis; shoulder | L92005 | ICPC 2 PLUS |
|  | Capsulitis; adhesive | L92008 | ICPC 2 PLUS |
|  | Capsulitis; shoulder | L92009 | ICPC 2 PLUS |
|  | Frozen shoulder | L92010 | ICPC 2 PLUS |
|  | Tenosynovitis; shoulder | L92014 | ICPC 2 PLUS |
|  | Epicondylitis; shoulder | L92015 | ICPC 2 PLUS |
| **Total rotator cuff related pain/injuries** |  |  |  |
|  | Bursitis; shoulder | L92001 | ICPC 2 PLUS |
|  | Rotator cuff syndrome | L92003 | ICPC 2 PLUS |
|  | Tendonitis; shoulder | L92013 | ICPC 2 PLUS |
|  | Tendonitis; supraspinatus | L92016 | ICPC 2 PLUS |
|  | Painful arc syndrome | L92017 | ICPC 2 PLUS |
|  | Injury; rotator cuff | L79041 | ICPC 2 PLUS |
|  | Injury; supraspinatus | L81023 | ICPC 2 PLUS |
|  | Tear; supraspinatus | L81028 | ICPC 2 PLUS |
| **Group** | **Term** | **Code** | **Classification system** |
| **Medications** |  |  |  |
| **Opioids** | Opioids | N02A | ATC |
| **Panadol** | Panadol | N102 | CAPS |
| **NSAID** | NSAID | M1 | CAPS |
| **Steroid Injection** | Betamethasone systemic injection | H201 | CAPS |
|  | Methylprednisolone injection | H205 | CAPS |
|  | Hydrocortisone injection | H208 (inj) | CAPS |
|  | Steroid injection NEC | H213 | CAPS |
|  | Cortisone injection NEC | H214 | CAPS |
| **Steroid - oral** | Prednisone | H202 | CAPS |
|  | Predsolone | H203 | CAPS |
|  |  |  |  |
| **Advice/education/ counselling** |  |  |  |
|  | Observe/health education/advice | -45 (ex observe/ wait) | ICPC-2 PLUS |
|  | Therapeutic counselling/listening | -58 | ICPC-2 PLUS |
| **AEC exercise** |  |  |  |
|  | Advice/education; exercise | A45004 | ICPC-2 PLUS |
|  | Counselling; exercise | A58005 | ICPC-2 PLUS |
| **AEC medication** |  |  |  |
|  | Advice/education; medication | A45015 | ICPC-2 PLUS |
|  | Advice/education; Dorsette box | A45032 | ICPC-2 PLUS |
|  | Advice/education; Webster pack | A45033 | ICPC-2 PLUS |
| **Physical medicine** | Physical medicine/rehabilitation | -57 | ICPC-2 |
| **Surgeon** |  |  |  |
|  | Referral; surgeon | A67003 | ICPC-2 PLUS |
|  | Referral; orthopaedic surgeon | L67002 | ICPC-2 PLUS |
|  |  |  |  |
